# Correction: A study of the structural properties of sites modified by the O-linked 6-N-acetylglucosamine transferase

**DOI:** 10.1371/journal.pone.0190461

**Published:** 2017-12-27

**Authors:** Thiago Britto-Borges, Geoffrey J. Barton

The enzyme name “O-linked β-N-acetylglucosamine” is incorrect in the article title, as well as in the second sentence of the Introduction section. In both of these cases, “O-linked 6-N-acetylglucosamine” should be “O-linked β-N-acetylglucosamine”. The correct title is: A study of the structural properties of sites modified by the O-linked β-N-acetylglucosamine transferase. The correct citation is: Britto-Borges T, Barton GJ (2017) A study of the structural properties of sites modified by the O-linked β-N-acetylglucosamine transferase. PLoS ONE 12(9): e0184405. https://doi.org/10.1371/journal.pone.0184405.
